# Dispersibility of vapor phase oxygen and nitrogen functionalized multi-walled carbon nanotubes in various organic solvents

**DOI:** 10.1038/srep26208

**Published:** 2016-05-18

**Authors:** Maryam Khazaee, Wei Xia, Gerhard Lackner, Rafael G. Mendes, Mark Rümmeli, Martin Muhler, Doru C. Lupascu

**Affiliations:** 1 Institute for Materials Science and Center for Nanointegration Duisburg-Essen (CENIDE), University of Duisburg-Essen, Universitätsstraße 15, 45141 Essen, Germany; 2 Laboratory of Industrial Chemistry, Ruhr-University Bochum, 44780 Bochum, Germany; 3 Leibniz Institute for Solid State and Materials Research (IFW) Dresden, Helmholtzstraße 20, D-01069 Dresden, Germany; 4 Center of Polymer and Carbon Materials, Polish Academy of Sciences, M. Curie-Sklodowskiej 34, Zabrze 41-819, Poland; 5 College of Physics, Optoelectronics and Energy & Collaborative Innovation Center of Suzhou Nano Science and Technology, Soochow University, Suzhou 215006, China

## Abstract

The synthesis and characterization of gas phase oxygen- and nitrogen-functionalized multi-walled carbon nanotubes (OMWCNTs and NMWCNTs) and the dispersibility of these tubes in organic solvents were investigated. Recently, carbon nanotubes have shown supreme capacity to effectively enhance the efficiency of organic solar cells (OSCs). A critical challenge is to individualize tubes from their bundles in order to provide homogenous nano-domains in the active layer of OSCs. OMWCNTs and NMWCNTs were synthesized via HNO_3_ vapor and NH_3_ treatments, respectively. Surface functional groups and the structure of the tubes were analyzed by temperature-programmed desorption, Fourier transform infrared spectroscopy, transmission electron microscopy, and Raman spectroscopy which confirmed the formation of functional groups on the tube surface and the enhancement of surface defects. Elemental analysis demonstrated that the oxygen and nitrogen content increased with increasing treatment time of the multi-walled carbon nanotube (MWCNT) in HNO_3_ vapor. According to ultra-violet visible spectroscopy, modification of the MWCNT increased the extinction coefficients of the tubes owing to enhanced compatibility of the functionalized tubes with organic matrices.

Multi-walled carbon nanotubes (MWCNTs) are composed of concentrically rolled graphene sheets and demonstrate remarkable optical, mechanical, and electrical properties^1^,^2^, which make them promising candidates for various applications. However, the high aspect ratio of carbon nanotubes (CNTs) and the existence of strong π-π interaction between tubes (0.5–2 eV/nm) in vacuum give them a hydrophobic nature^3^,^4^, which hinders their solubility in aqueous and related solvents. As a result, they have the tendency to aggregate in these media. Modification of the carbon nanotube surface can donate them the capability to interact with an organic environment via affecting the CNT surface energy^5^,^6^. Moreover, it can alter the CNT energy levels with respect to vacuum energy (E_vac_) and as a consequence, e.g. their role as acceptor or donor species in the active layer of organic solar cells^7^,^8^,^9^.

Substantial efforts have been carried out to functionalize graphene and CNTs with oxygen species including liquid-phase acid treatment of CNTs^10^,^11^, plasma purification and oxidation^12^, and gas phase oxidation^13^,^14^. Although modified oxygen groups on CNTs via direct acid treatment improve their compatibility in solvents, albeit, such liquid phase oxidation severely deteriorates the crystalline structure of CNTs and their electrical conductivity. Among the above mentioned methods for CNT oxidation, gas phase oxidation of CNTs is a promising one, which can create low damage to CNTs^13^. This alternative oxygen modification method exploits the covalent interaction of nitric acid with CNTs in the gas phase. Moreover, nitrogen doping of the graphitic surface of CNTs, as a facile and effective doping methodology, preserves the high electrical conductivity of the tubes^15^,^16^. This phenomenon along with a significant difference between the electronegativity of N and C atoms gives rise to a reduced work function^17^. Figure 1 depicts a schematic of functionalized tubes and several modified groups on CNT surfaces after HNO_3_ (a) and NH_3_ (b) treatments^18^,^19^.

Low carrier mobility of organic materials is an obstacle in organic solar cells (OSCs). In recent years, utilizing CNTs in bulk heterojunction (BHJ) solar cells has attracted much attention due to their superior electrical properties^20^. For this purpose, proper individualization of CNTs in organic solvents is indispensable, because it facilitates their incorporation into photovoltaically active matrices providing nano-domains in the BHJ. The entity of these nano-domains in the active layer may exceed exciton dissociation, because these domains provide nano-boundaries in the order of the exciton diffusion length (10–20 nm)^21^. In addition, the out of plane unhybridized P_z_ orbitals of CNTs form an appropriate pathway for transporting charge to the electrodes^22^. These effects can enhance solar cell efficiency^23^.

As most organic mediating materials have insulating properties, it is essential to remove them for electronic applications. Elimination of solvents from CNTs is achievable by facile heating treatment to enable direct interaction between CNTs and BHJ active materials. In the present article, covalent modification of MWCNTs was studied using oxygen and nitrogen in the gas phase. Further investigations were performed to observe the behaviour of modified and unmodified tubes in some organic solvents. This research aims to find out the nature of chemical doping of MWCNTs and their dispersion capability in organic solvents. Thoroughly understanding these phenomena is essential and beneficial for their subsequent usage e.g. in BHJ solar cells.

## Results and Discussion

**Figure 2:**
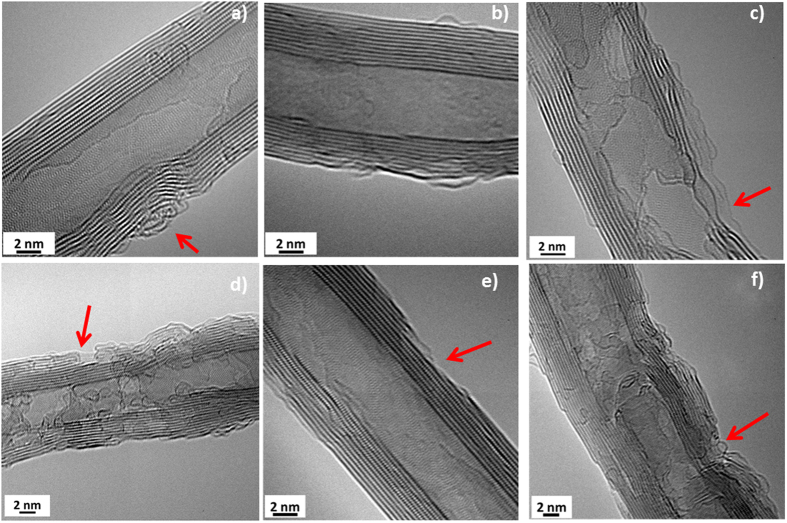
HRTEM images of pristine MWCNTs (**a**), purified MWCNTs (**b**), OMWCNT-48 (**c**), OMWCNT-72 (**d**), NMWCNT-48 (**e**) and NMWCNT-72 (**f**). The red arrows indicate the presence of amorphous carbon on pristine MWCNT (**a**) and the created defects on the functionalized tubes.

Figure 2a–f depicts high-resolution TEM (HRTEM) images of the pristine MWCNT (a), purified MWCNT (b), OMWCNT-48 (c), OMWCNT-72 (d), NMWCNT-48 (e), and NMWCNT-72 (f). Clearly, most of the pristine and purified tubes are free from metal contaminations (>95% carbon purity) and there are a limited number of defects on the tube sidewalls. In addition, amorphous carbon wrapped around pristine and purified tubes. The purification process was not deleterious for the pristine tubes. However, functionalization of tubes by oxygen and nitrogen resulted in the creation of a bumpy morphology in all of the functionalized tubes which may be attributed to the introduction of new defects on the CNT sidewalls. The red arrows in Fig. 2 highlight the locations of amorphous carbon and sidewall damages in the pristine and functionalized tubes, respectively. In addition, the amount of amorphous carbon was enormously decreased on the functionalized tubes sidewall, as compared with pristine MWCNT. It can be concluded that oxygen and nitrogen functionalization of MWCNT leads to the removal of amorphous carbon from the tube sidewall and introduce some defects along the CNT surface.

These defect sites could enhance the susceptibility of the tubes for interacting with other functional groups. Chemical composition of functionalized tubes was determined applying EDX inside the TEM column (see Supplementary Fig. S1) to provide insights into the concentration of trace metal impurities. Owing to the vapor phase oxygen modification of the tubes, trace of oxygen can be seen in all the EDX spectra. The spectra indicate the presence of significant amount of copper (Cu) which is related to the TEM grid. However no peak appeared regarding the metal contaminations verifying high purity of the tubes.

**Figure 3:**
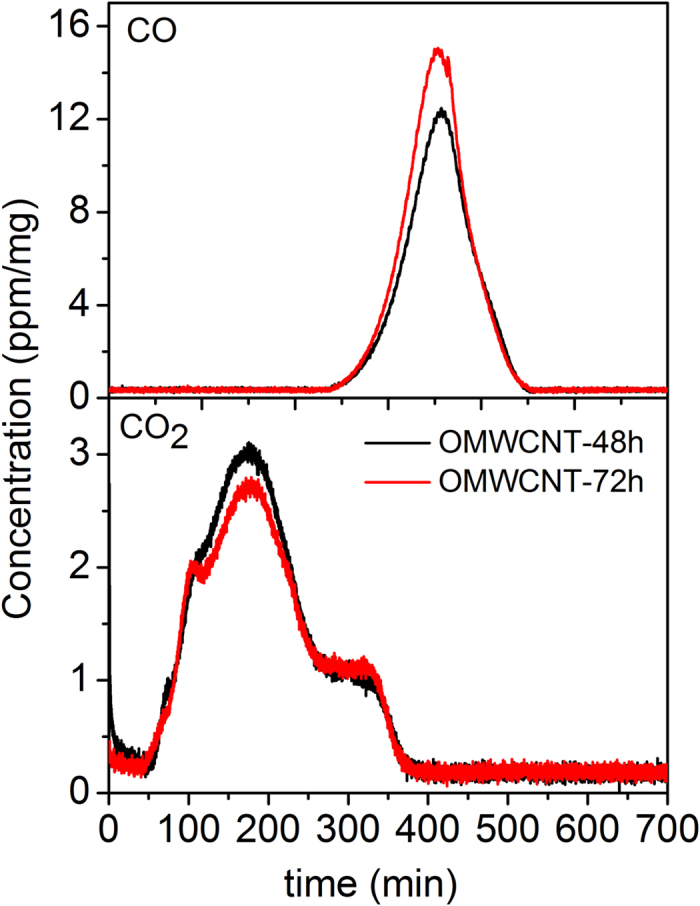
CO and CO_2_ profiles of oxygen vapor treated MWCNTs for 48 hours (red) and 72 hours (black) performed in helium at a heating rate of 2 °C min^−1^.

In order to examine the type, amount, and thermal stability of surface oxygen groups which were produced during gas phase oxidation of purified MWCNTs (OMWCNTs-48 h and OMWCNTs-72 h), TPD experiments were carried out. Figure 3 depicts CO and CO_2_ desorption profiles obtained upon heating OMWCNTs from room temperature to 1000 °C at a heating rate of 2 °C min^−1^. The notable desorption of CO and CO_2_ from the oxygen functionalized tubes shows that a considerable amount of surface oxygen groups was created by HNO_3_ vapor treatment. The amount of released CO_2_ and CO from OMWCNTs was determined by quantitative analysis of TPD results (Table 1). These results indicate that the amount of released CO_2_ did not increase by extending the oxygen modification time from 48 h to 72 h, whereas the amount of CO rose remarkably from 1.3 mmol g^−1^ to 1.61 mmol g^−1^. This can be attributed to the type of functionalized groups on OMWCNT surfaces. A small surface area of the tubes limits the incorporation of large functional groups consisting of carboxylic groups and carboxylic anhydrides onto the tube surface which are the origin of desorbed CO_2_. On the other hand, the increase in the amount of desorbed CO confirms the existence of more small functional groups on the tubes, since CO mainly originates from the decomposition of hydroxyl, carbonyl, and ether groups^24^. Therefore, it appears that by increasing treatment time in HNO_3_ vapor, more phenol and ether groups are created on the sidewalls of the tube than carboxylic acid groups.

**Figure 4:**
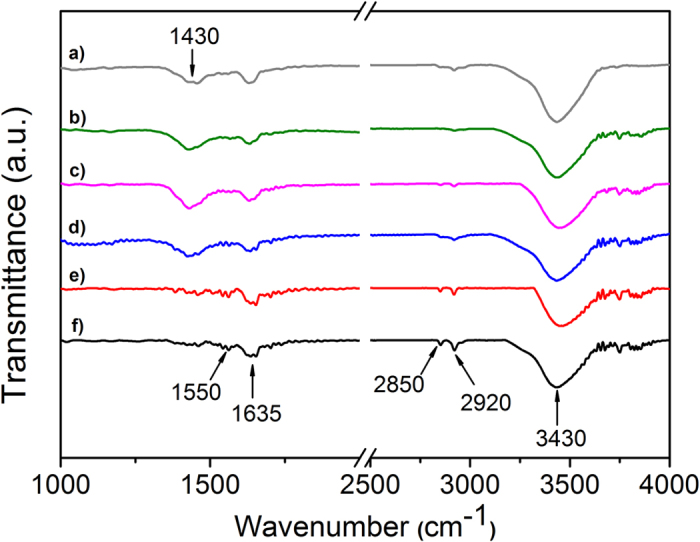
FTIR spectra of (a) NMWCNT-72, (b) NMWCNT-48, (c) OMWCNT-72, (d) OMWCNT-48, (e) PMWCNTs and (f) as-received MWCNTs.

IR spectroscopy was accomplished to specify the functional groups on the CNT surface (Fig. 4). The spectra of a) NMWCNT-72, b) NMWCNT 48, c) OMWCNT72, d) OMWCNT-48, e) PMWCNTs, and a) as-received MWCNTs were collected. In these spectra, the dominant transmittance at 3430 cm^−1^ was assigned to O-H stretching vibration which could be due to absorbed water from the environment, O-H or carboxylic groups on CNT surfaces. In the spectrum of the as-received MWCNT, the small peaks around 2920 cm^−1^ and 2850 cm^−1^ are consistent with asymmetric and symmetric CH_2_ stretching vibration bands, respectively^25^.

These functional groups could be formed at defect sites of the MWCNT surface. In the spectra of OMWCNTs and NMWCNTs, these peaks are diminished. This can be correlated to the removal of chemisorbed hydrogen from MWCNT surface during tube functionalization and generation of some other functional groups on the defect sites. The bands in the 1750–1550 cm^−1^ range can be assigned to C=O or C=C groups. As a consequence, the bands around 1635 cm^−1^ and 1550 cm^−1^ in these spectra are correlated to the carboxylic groups and aromatic and unsaturated structure of >C=C< bonds. The characteristic band of C-O appears near 1430 cm^−1^ in these spectra. As it is depicted, the intensity of this peak intensifies with oxidation of the MWCNTs implying modification of the oxygen groups on the CNT surface, whereas the intensity of this feature decreases by nitrogen doping of OMWCNTs emphasizing the decomposition of surface oxygen groups from the surface of the tubes. No distinct peak was observed related to the nitrogen groups on the CNT surface which is in good agreement with the result. This phenomenon may be due to the existence of only a small amount of nitrogen in the functionalized tubes. Therefore, elemental analysis was performed to investigate the average amount of modified oxygen and nitrogen groups to the CNTs surface.

Furthermore, elemental analysis was carried out to measure the amount of carbon (C), nitrogen (N), hydrogen (H), and oxygen (O) in these tubes (Table 2). The elemental percentages represent the means from two independent measurements. Carbon was obviously the most abundant element observed in all CNTs. The highest level of carbon was detected in as-received MWCNT (98.75 wt%) then in purified tubes (98.2 wt%). A little decrease in the carbon content in the purified tubes can be attributed to the removal of amorphous carbon from the surface of the tubes. It can be seen from Table 2 that the content of carbon reduced by oxygen and nitrogen doping of the tubes. Low trace (< 0.2 wt%) of detected hydrogen in all the samples suggests low levels of adsorbed hydrogen gas and hydrogen-terminated carbon atoms. In addition, the highest content of oxygen was detected in the tubes treated by vapor HNO_3_ providing oxygen modified tubes (OMWCNTs). The observed reduction in the oxygen content of nitrogen functionalized tubes can be ascribed to the decomposition of oxygen groups from OMWCNTs and doping of the tubes by nitrogen groups.

Moreover, traces of nitrogen were observed in the samples treated for 6 h under flowing NH_3_. As the nitrogen content enhanced from 0.89 wt% to 1 wt% by increasing the treatment time of OMWCNT from 48 h to 72 h, it can be concluded that the content of nitrogen on NMWCNT prepared by post-treatment of OMWCNT depends on the treatment time of MWCNT in HNO_3_ vapor.

**Figure 5:**
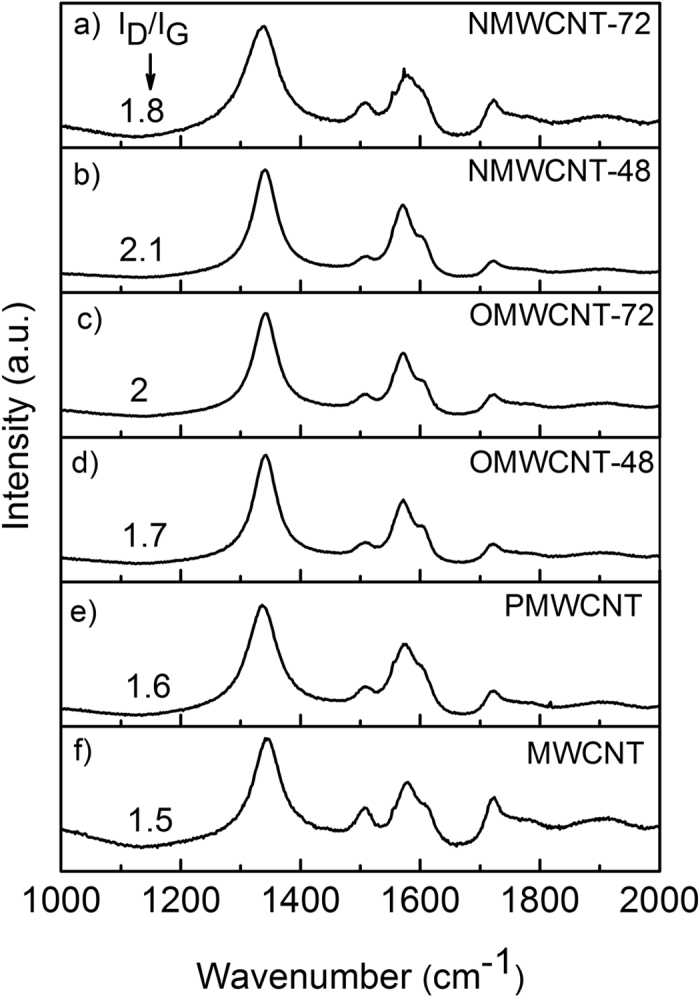
Raman spectra of NMWCNT-72 (**a**), NMWCNT-48 (**b**), OMWCNT-72 (**c**), OMWCNT-48 (**d**), PMWCNT (**e**), and MWCNT (**f**). The D-band and G-band were observed at 1335 cm^−1^ and 1576 cm^−1^, respectively.

Figure 5 represents the Raman spectra of as-received MWCNTs, PMWCNT, and the functionalized tubes. The two main characteristic bands presented in all the spectra at 1335 cm^−1^ and 1576 cm^−1^ can be assigned to the D-band and the G-band, respectively. The existence of amorphous carbon and any defects on the tubes result in the D-band as a double-resonance Raman mode. Moreover, the G-band is related to the crystallinity of the tubes. The Raman spectrum of MWCNT (see Supplementary Fig. S2) shows multiple splitting of G-band mode. A weak peak at 1506 cm^−1^ and a strong peak at 1576 cm^−1^ with two shoulders at 1557 cm^−1^ and 1607 cm^−1^ can be seen in the spectrum f. Interlayer interactions are likely inducing such graphite-like behavior in the tubes^26^.

The intensity ratio of the D-band to the G-band in this figure provides information regarding the produced structural disorder in the tubes due to various surface treatments. The higher I_D_/I_G_ of the functionalized tubes as compared to that of pristine tubes suggests a higher number of defects, which has modified the tubes and altered the sp^2^ hybridization of the carbon atoms in the graphene layer to sp^3^ hybridization.

**Figure 6:**
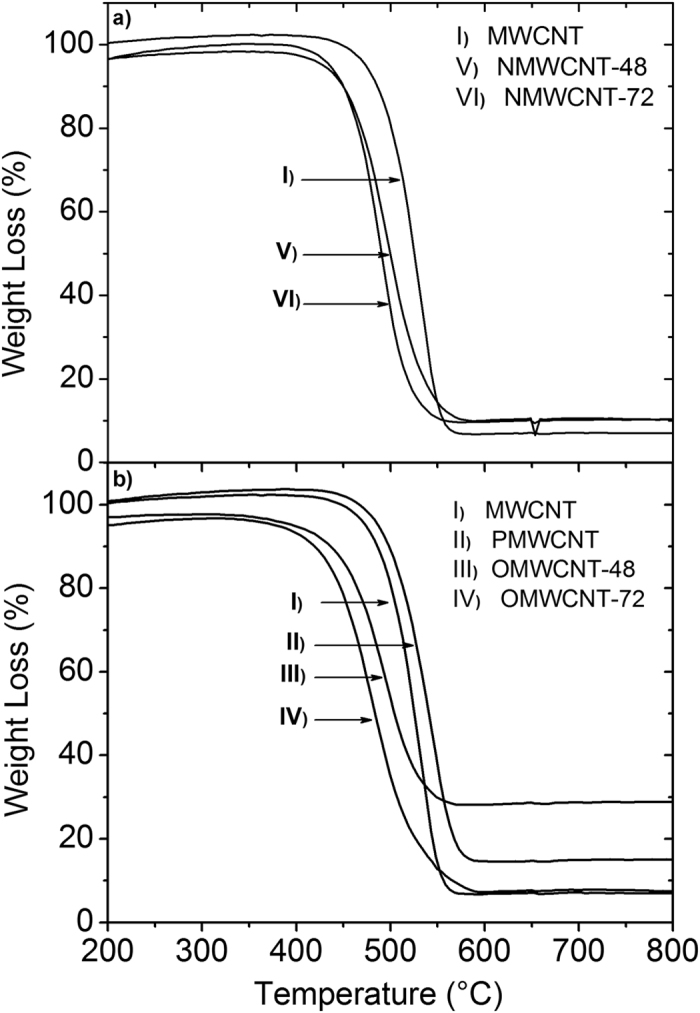
TGA curves of pristine MWCNT (I), PMWCNT (II), OMWCNT-48 (III), OMWCNT-72 (IV) (**a**) and NMWCNT-48 (V), and NMWCNT-72 (VI) (**b**).

Thermal stability of CNTs tremendously depends on the degree of structural order^25^. Defect sites on the tubes are the sources of faster corrosion of the tubes. As a consequence, TGA is an appropriate methodology to characterize the structural order of CNTs. Figure 6 in a,b shows the thermogravimetric analysis of the as-received, purified, and functionalized tubes presenting the thermal stability of each tube during heating. Since the oxidation of carbon happens at temperatures higher than 200 °C, the graphs were normalized to this temperature. At lower temperatures, the absorbed water and organic impurities are removed from the tube walls^27^. The TGA curve of the as- received MWCNT (Fig. 6(I)) shows that the oxidation of the pristine tubes begins at 493.3 °C and reaches to a maximum weight loss rate at 533.2 °C. However, the initiation and oxidation temperatures of the functionalized tubes are lower than those of pristine tubes. As can be seen from Fig. 6(II) and Supplementary Table S1, after the pristine MWCNTs were purified, the initiation and oxidation temperatures increase compared to pristine tubes. It is ascribed to the removal of catalyst particles and amorphous carbon from the tube wall. However, the initiation and oxidation temperatures of the functionalized MWCNTs decrease noticeably. This behavior can be interpreted by referring back to the TEM figures. Premier stability of pristine MWCNTs and PMWCNTs than modified tubes toward high temperature oxidation is due to the existence of fewer defects in the tubes. Functionalization of CNT via vapor phase HNO_3_ and NH_3_ treatments alters the hexatomic structure of the tubes via modification of the chemical structure of the tubes. The added defects to the CNT surface decrease the oxidation stability of the tubes. Furthermore, it is feasible to estimate the residual mass for each sample from the weight loss curves after complete combustion of the tubes at 800 °C (see Supplementary Table S1). The calculated residual mass attributes to the amount of metal oxides remnants of each tube. Since some carbon is removed during purification and modification of the tubes, a higher residual mass is observed owing to the higher catalyst residual.

**Figure 7:**
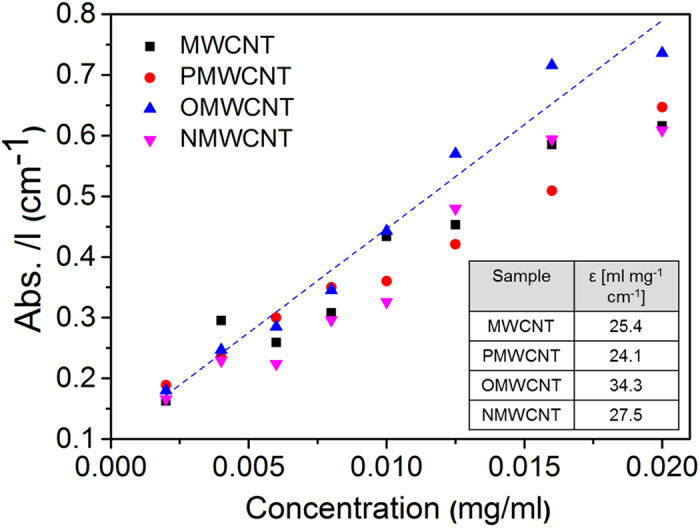
Absorbance per optical length measured at λ = 500 nm, A_500_/l, before centrifugation as a function of nanotubes concentrations. The dashed line depicts the fitted line to the scattered values of OMWCNT. The inset table shows the calculated extinction coefficient for the tubes.

In addition, 1,2 dichlorobenzene was selected as a proper solvent for the dispersion of pristine MWCNT, PMWCNT and functionalized tubes. UV-Vis spectra were collected for each of the diluted samples (see Supplementary Fig. S3) and absorbance at 500 nm, A_500_, was measured and then divided to the optical length, l, to give A_500_/l. These data are plotted versus initial concentration of CNTs (Fig. 7). Based on the Beer-Lambert law, the slopes of these graphs provide extinction coefficients for each type of tube. The inset table in Fig. 7 depicts the variation of the extinction coefficients due to functionalization. MWCNTs and purified ones show the lowest extinction coefficients attributing to their low solubility in the organic solvent. Moreover, enhancement in the extinction coefficients of the oxidized tubes (34.3 ml mg^−1^ cm^−1^) confirms that oxygen functionalization of MWCNT improved the solubility of the tubes in the organic solvent. From these values, it is evident that the OMWCNT has the highest degree of dispersibility in the organic solvent compared to the other tubes. However, no considerable improvement is observed for the extinction coefficient of NMWCNTs (27.5 ml mg^−1^ cm^−1^). It can be concluded that the type of functional group can determine the solubility of the tubes in the organic solvents. As a consequence, it can be inferred that the CNT structure, solubility, amount of defects, and type of functional groups influence the extinction coefficient which is in agreement with previous research for SWCNTs^28^.

The hydrophobic nature of the CNTs and the strong van-der-Waals interaction between tubes hinder their solubility in most solvents. Utilizing CNTs in BHJs solar cells raises concerns with regards to appropriate individualization of tubes in order to provide convenient nano-domains in BHJ.

**Figure 8:**
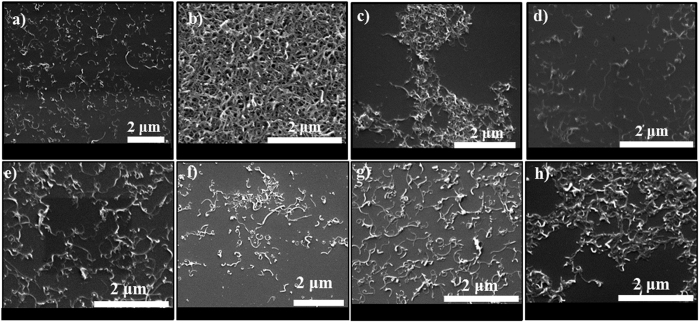
SEM images of dispersed MWCNTs in (**a**) 1,2 dichlorobenzene, (**b**) benzyl alcohol, (**c**) dimethylformamide and (**d**) chloroform. SEM images of dispersed PMWCNT in (**e**) 1,2 dichlorobenzene, (**f**) benzyl alcohol, (**g**) chlorobenzene and (**h**) chloroform deposited on Si wafer.

Supplementary Fig. S4 shows the digital photographs of supernatants of sonicated 1 mg ml^−1^ CNTs in different organic solvents followed by 1 h centrifugation at 10 k rpm. The supernatants of tubes were collected after centrifugation. A summary of the dispersion ability of these solvents was depicted in Supplementary Table S2. As this figure shows, a huge amount of MWCNTs are transferred to the organic phase when benzyl alcohol, 1,2 dichlorobenzene, chloroform, and dimethylformamide were utilized. NMWCNT-48 was sonicated in the same organic solvents. However, in the chloroform and chlorobenzene-NMWCNT solutions, phase separation between organic solvent and tubes is observed ascribed to the incompatibility of tubes in these organic solvents. Therefore, no further centrifugation was performed for these two solutions. Among these solutions, the samples with bright colors confirm no compatibility of the tubes with the solvents. In order to visualize the quality of dispersion, SEM was performed. The SEM images of pristine MWCNTs and purified MWCNTs dispersed in various organic solvents are shown in Fig. 8. As evidenced by SEM (Fig. 8a–d), pristine MWCNTs could be dispersed properly in 1,2 dichlorobenzene and chloroform. However, they aggregate in benzyl alcohol and dimethylformamide.

**Figure 9:**
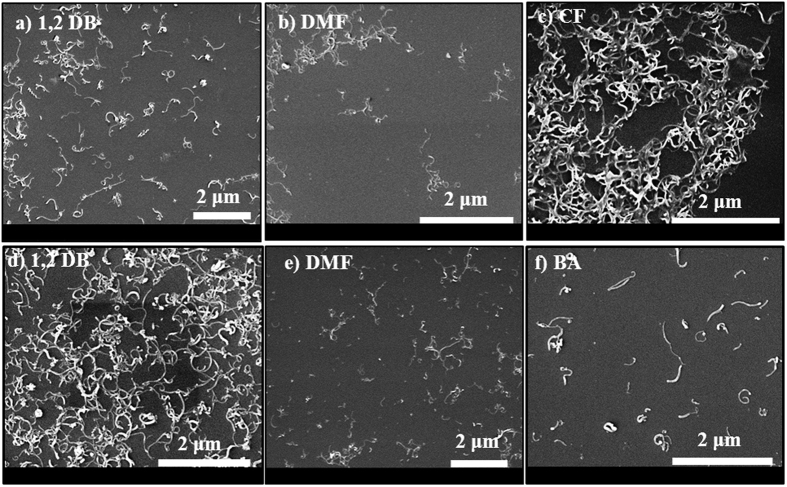
SEM images of dispersed oxidized MWCNTs in (**a**) 1,2 dichlorobenzene, (**b**) dimethylformamide and (**c**) chloroform. Second row: SEM images of dispersed nitrogen doped MWCNT in (**d**) 1,2 dichlorobenzene, (**e**) dimethylformamide and (**f**) benzyl alcohol deposited on Si wafer.

The same experimental procedures were used for dispersion of purified MWCNTs in organic solvents. SEM images of these samples (Fig. 8e–h) display how PMWCNTs exfoliated in different organic solvents. From these images, it can be deduced that all of these solvents have appropriate ability to individualize PMWCNTs. OMWCNT-48 was utilized to investigate the compatibility of oxygen functionalized tubes with an organic phase. Digital photographs of all the supernatants were collected (Supplementary Fig. S3). Among these supernatants DMF-OMWCNT depicts dark black color emphasizing the existence of a large amount of tubes in the solution. In addition, the SEM images (Fig. 9) depict that the tubes modified by oxygen and nitrogen were properly dispersed in 1,2 dichlorobenzene and dimethylformamide. Moreover, chloroform and benzyl alcohol could appropriately disperse oxygen and nitrogen functionalized tubes, respectively. This study thus determines the dispersibility of pristine and functionalized CNTs in various organic solvents, which facilitates their incorporation into polymer matrics of OSCs.

In this work, oxygen and nitrogen modification of MWCNT were performed in the gas phase. The increase in the treatment time of MWCNT in HNO_3_ vapor resulted in the increase of oxygen content as well as enhancement in the nitrogen content. FTIR and EDX analyses demonstrated that oxygen-containing groups were attached to the treated tubes. Furthermore, SEM observations depicted the dispersibility of the pristine, purified and functionalized tubes in different organic solvents. TEM and Raman studies revealed that functionalization of the tubes generated more defects on the tube sidewall. Creation of these defects on the tubes diminished the thermal stability of the functionalized tubes, as disclosed by TGA. Oxygen modification of MWCNT ameliorated the extinction coefficients of the tubes as compared to that of pristine, purified, and nitrogen doped ones.

## Methods

### Materials and Methods

Organic solvents consisting of benzyl alcohol (BA), 1,2 dichlorobenzene (1,2 DB), chlorobenzene (CB), chloroform (CF), and dimethylformamide (DMF) were purchased from Sigma Aldrich and utilized as-received. MWCNTs (Baytubes® C 150 P) in powder form with outer diameters 13–16 nm were obtained from Bayer AG, Leverkusen, Germany. CNT purification was performed by washing them in 1.5 M HNO_3_ under stirring for 48 h at room temperature in order to eliminate residual growth catalysts. The obtained purified tubes were entitled as PMWCNTs. PMWCNTs were modified by oxygen (OMWCNTs) via HNO_3_ vapor at 200 °C for 48 h (OMWCNT-48) and 72 h (OMWCNT-72). Nitrogen-containing functional groups on MWCNTs (NMWCNTs) including NMWCNTs-48 and NMWCNTs-72 were synthesized by post-treatment of OMWCNTs-48 and OMWCNTs-72, respectively. For this purpose, 50 mg OMWCNTs are loaded into a tubular reactor and treated at 400 °C for 6 h under flowing NH_3_ (10% NH_3_ in He, 50 ml min^−1^) at a flow rate of 50 sccm^13^.Dispersion capability of all aforementioned organic solvents for CNT dispersion including as-received MWCNTs, PMWCNTs, OMWCNTs-48 and NMWCNTs-48 was investigated by preparing 1 mg ml^−1^ of these MWCNTs in the organic solvents and tip sonicated (Bandelin, Sonopuls HD 70, Max. power: 60 W) for 15 min at 20% amplitude in an ice bath. The sonicated solutions were ultracentrifuged (Mikro 220 R, Hettich, Germany) at 10 k rpm for 1 hour to elicit larger aggregates from the dispersion. The supernatants were attentively decanted for further characterization.

### Characterization

Magnified views of the as-received, purified and functionalized tubes were provided using an aberration-corrected high resolution TEM at an accelerating voltage of 80 keV (FEI Titan TEM). For this purpose, a small amount of the powder containing CNTs was directly pressed onto TEM copper grids and imaged. The low voltage minimizes damage to the CNT walls by the electron beam during exposure. The energy-dispersive X-ray (EDX) measurements of the functionalized tubes were conducted inside the TEM column. To guarantee the reproducibility of the results, imaging and EDX spectra were collected in various regions of each sample. In order to investigate the nature of the oxygen functional groups on OMWCNT surfaces, temperature-programmed desorption (TPD) measurements were carried out in a horizontal quartz reactor with an inner diameter of 4 mm. An online infrared detector (Bühler Technologies, Germany) was employed to quantitatively analyze the released CO and CO_2_ during the decomposition of oxygen groups from OMWCNT surfaces. OMWCNT was heated under flowing helium (99.9999%, 30 ml min^−1^) from room temperature to 1000 °C at a heating rate of 2 °C min^−1^. The temperature was kept constant for 2 hours at 1000 °C before cooling down to room temperature.

The modified functional groups on the tubes were characterized by applying Fourier transform infrared spectroscopy (FTIR). IR spectra were measured in a Bruker VERTEX 70 spectrometer in transmittance mode by grinding a very low concentration of CNTs into potassium bromide. Furthermore, the elemental analysis (CHN/O) was done by elemental analyzer EuroVector EA 3000. Thermogravimetric analysis (TGA) experiments were fulfilled using a NETZSCH STA 409 PC Luxx® to represent the thermal stability and purity of the different tubes by characterizing the weight loss of tubes during heating. In each experiment, carbon nanotubes (5 mg) were heated from 30 °C to 1000 °C at a heating rate of 5 K/min under O_2_ flow. Raman spectra were acquired using Renishaw, inVia Raman microscope with a laser operating at 532 nm. The intensity ratio of I_D_ to I_G_ was calculated based on the spectra to estimate the structural changes in the tubes as a result of tube modification. Ultra-Violet Visible (UV-Vis) spectroscopy (Shimadzu UV-2600 spectrophotometer, wavelength range: 300–900 nm) was carried out to characterize the extinction coefficient of the tubes. Characterization of MWCNT concentration after centrifugation can be achieved by applying the Beer-Lambert law:


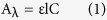


where A_λ_ is the absorbance at a specific wavelength λ, ε is the extinction coefficient, l is the optical pathway and C is the MWCNT dispersion concentration (mg ml^−1^). For this reason, the extinction coefficient of the MWCNTs was measured after sonication by dispersing 1 mg ml^−1^ CNTs in 1,2 dichlorobenzene and collecting the absorption values of diluted samples at 500 nm. Scanning electron microscopy (SEM; ESEM Quanta 400 FEG, FEI, Eindhoven, the Netherlands) was performed to visualize the dispersed MWCNTs. SEM samples were prepared by dropping 10 μl CNT suspensions on clean silicon substrates.

## Additional Information

**How to cite this article**: Khazaee, M. *et al.* Dispersibility of vapor phase oxygen and nitrogen functionalized multi-walled carbon nanotubes in various organic solvents. *Sci. Rep.* **6**, 26208; doi: 10.1038/srep26208 (2016).

## Supplementary Material

Supplementary Information

## Figures and Tables

**Figure 1:**
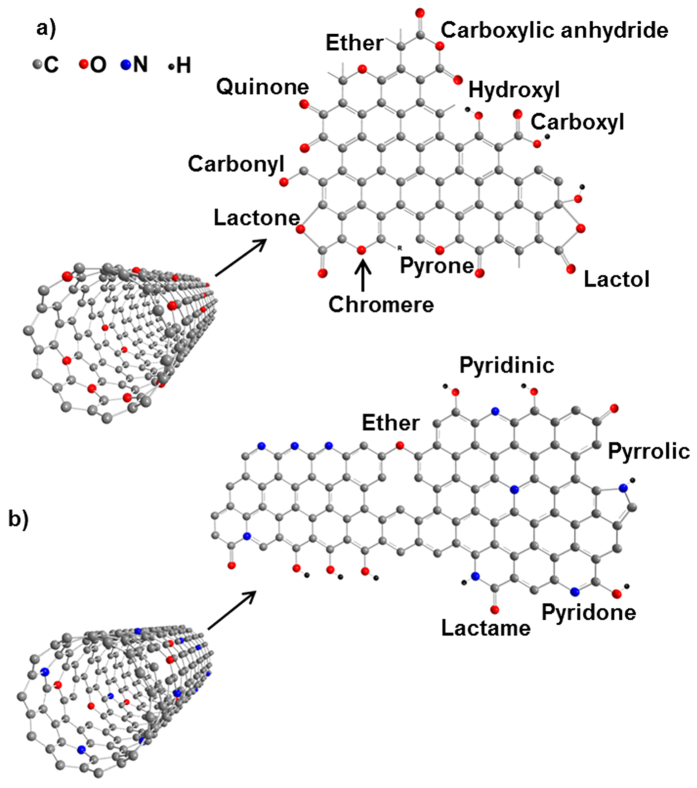
Schematic representation of oxygen doped MWCNT (**a**) and nitrogen doped MWCNT (**b**).

**Table 1:** Determined amount of desorbed CO_2_ and CO from TPD.

CNTs	CO_2_ (mmolg^−1^)	CO (mmol g^−1^)
OMWCNT-48	0.65	1.3
OMWCNT-72	0.643	1.61

**Table 2:** Elemental composition of various carbon nanotubes.

Tube	Elemental analysis (wt%)§			
	C	N	H	O
MWCNT	98.75	n.d.*	<0.1	<1
PMWCNT	98.2	n.d.	0.14	0.8
OMWCNT-48	91.9	n.d.	<0.1	5.55
OMWCNT-72	91.8	n.d.	<0.1	6
NMWCNT-48	95.3	0.89	0.13	2.58
NMWCNT-72	95.55	1	0.11	3.05

^§^The rest elements in the tubes could be metal impurity.

^*^n.d.: not detected element.
