# Supplementary material for: Dispersibility of vapor phase oxygen and nitrogen functionalized multi-walled carbon nanotubes in various organic solvents
Source: Sci Rep. 2016 May 18;6:26208. doi: 10.1038/srep26208 (PMC4870618; doi:10.1038/srep26208)
Supplement: Supplementary Information [file srep26208-s1.pdf]

Supplementary Information

Dispersibility of vapor phase oxygen and nitrogen functionalized multi-walled carbon nanotubes in various organic solvents

Maryam Khazaee[1,*], Wei Xia[2], Gerhard Lackner[1], Rafael G. Mendes[3], Mark Rümmeli[3,4], Martin Muhler[2], Doru C. Lupascu[1]

[1] Institute for Materials Science and Center for Nanointegration Duisburg-Essen (CENIDE), University of Duisburg-Essen, Universitätsstraße 15, 45141 Essen, Germany.

[2] Laboratory of Industrial Chemistry, Ruhr-University Bochum, 44780 Bochum, Germany.

[3] Leibniz Institute for Solid State and Materials Research (IFW) Dresden, Helmholtzstraße 20, D-01069 Dresden, Germany.

[4] Center of Polymer and Carbon Materials, Polish Academy of Sciences, M. Curie-Sklodowskiej 34, Zabrze 41-819, Poland.

* Maryam.khazaee@uni-due.de

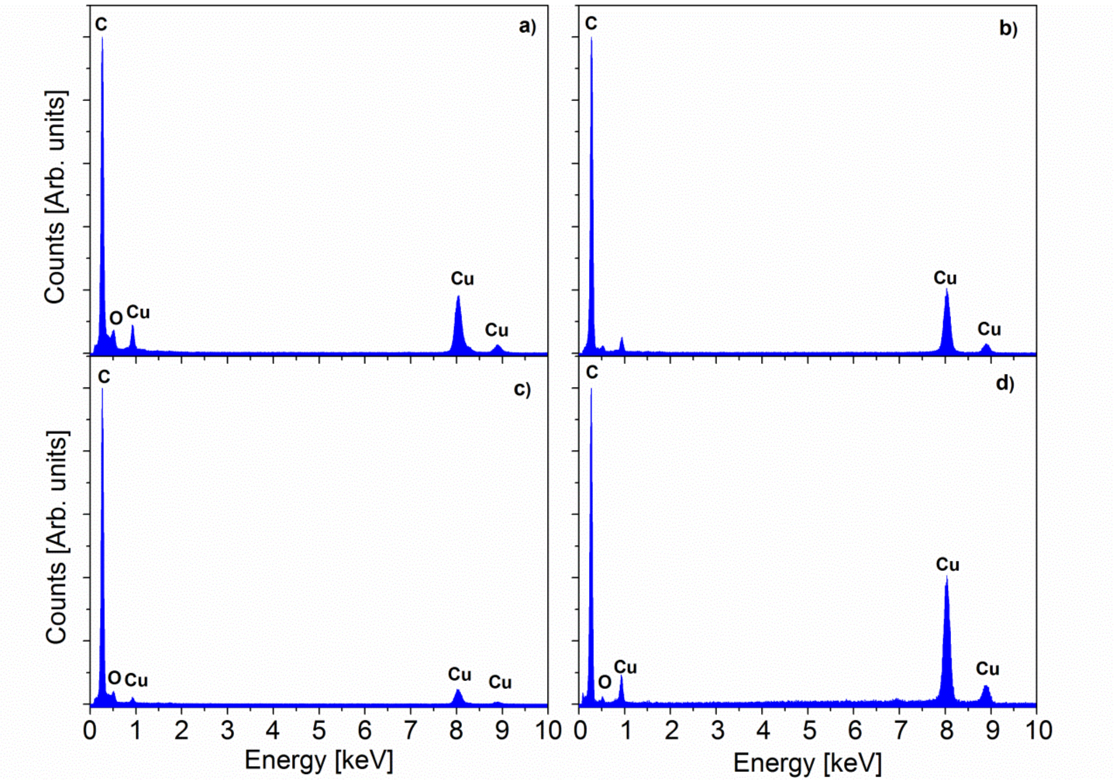

Supplementary Fig. S1. EDX spectra comparing the response of OMWCNT-48 (a), OMWCNT-72 (b), NMWCNT-48 (c) and NMWCNT-72 (d). The strong Cu peaks originate from the TEM Cu grid.

**Samples**	**Initiation T (°C)**	**Oxidation T (°C)**	**Residual mass percent (%)**
MWCNT	493.3	533.2	6.71
PMWCNT	500.6	542.95	14.57
OMWCNT-48	448.0	494.11	28.11
OMWCNT-72	435.4	479.37	7.37
NMWCNT-48	457.8	498.74	10.03
NMWCNT-72	456.0	488.84	9.06

Supplementary Table S1. The thermogravimetric parameters of analyzed CNTs.

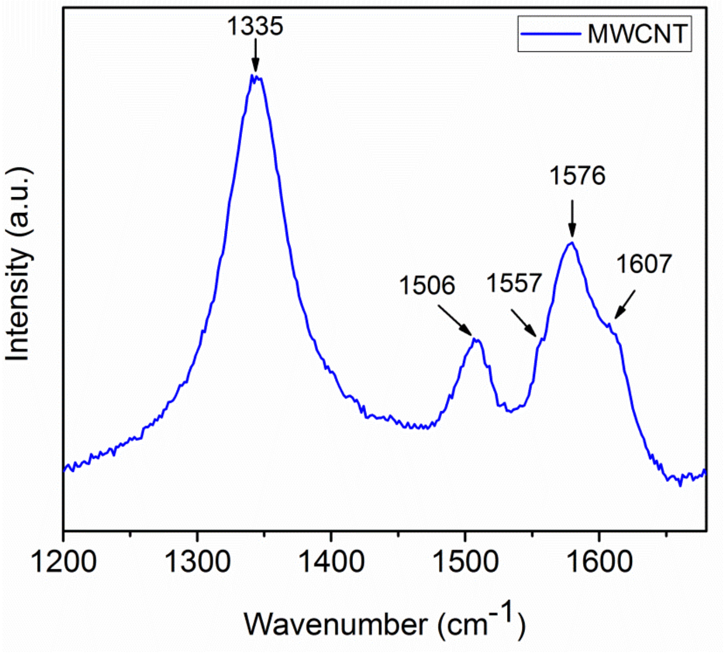

Supplementary Fig. S2. Raman spectrum of pristine MWCNT.

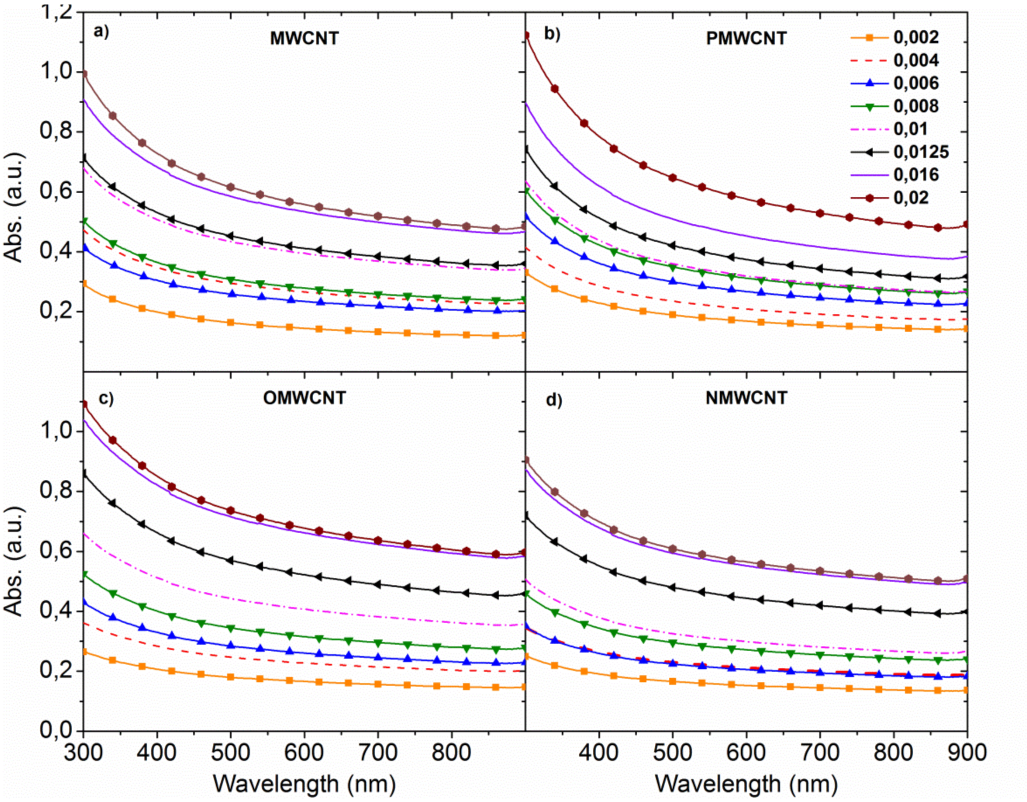

Supplementary Fig. S3. UV-Vis spectra of dispersed pristine MWCNTs (a), PMWCNT (b), OMWCNT-48 (c) and NMWCNT-48 (d) in 1,2 dichlorobenzene.

Tube	1,2 DB	BA	CF	CB	DMF
MWCNT	Yes	Yes	Yes	No	Yes
PMWCNT	Yes	Yes	Yes	Yes	No
OMWCNT-48	Yes	No	Yes	No	Yes
NMWCNT-48	Yes	Yes	No	No	Yes

Supplementary Table S2. Capability of different solvents for dispersion of CNTs.

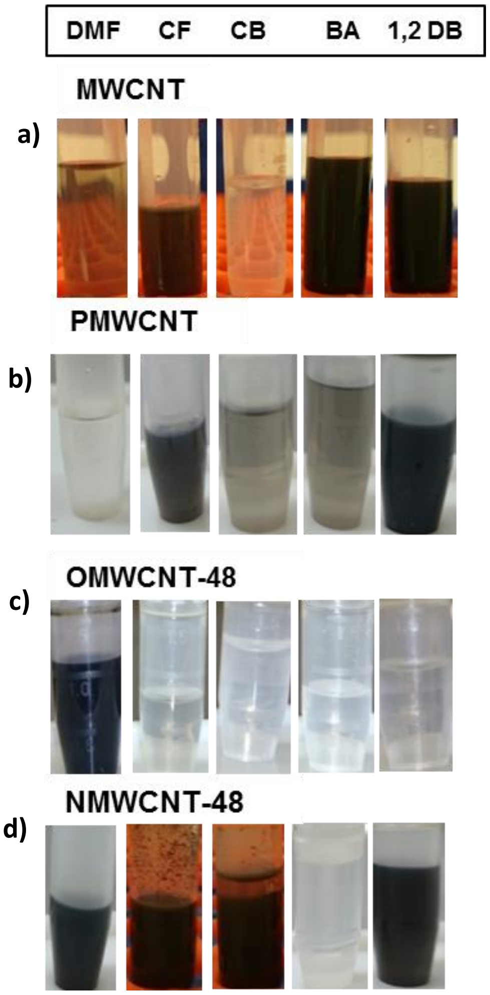

Supplementary Fig. S4. Digital photo images of a) MWCNT, b) PMWCNT, c) OMWCNT-48 and d) NMWCNT-48 in organic solvents (DMF, CF, CB, BA and 1,2 DB).
